# Comprehensive and safe school strategy during COVID-19 pandemic

**DOI:** 10.1186/s13052-021-00960-6

**Published:** 2021-01-09

**Authors:** Susanna Esposito, Nicola Cotugno, Nicola Principi

**Affiliations:** 1 Pediatric Clinic, Pietro Barilla Children’s Hospital, University of Parma, Via Gramsci 14, 43126 Parma, Italy; 2 Academic Department of Pediatrics (DPUO), Research Unit of Congenital and Perinatal Infections, Bambino Gesù Children’s Hospital, Rome, Italy; 3 Università degli Studi di Milano, Milan, Italy

**Keywords:** COVID-19, Mask, SARS-CoV-2, School, Telemedicine

## Abstract

**Background:**

Although several studies have tried to evaluate the real efficacy of school closure for pandemic control over time, no definitive answer to this question has been given. Moreover, it has not been clarified whether children or teenagers could be considered a problem for SARS-CoV-2 diffusion or, on the contrary, whether parents and school workers play a greater role. The aims of this review are to discuss about children’s safety at school and the better strategies currently able to reduce the risk of SARS-CoV-2 infection at school.

**Main aim:**

Compared to adults, very few cases of COVID-19 were diagnosed in children, who generally suffered from an asymptomatic infection or a mild disease. Moreover, school closure is systematically associated with the development of problems involving students, teachers and parents, particularly among populations with poor resources. Although several researches have tried to evaluate the real efficacy of school closure for pandemic control over time, no definitive answer to this question has been given. Available findings seem to confirm that to ensure adequate learning and to avoid social and economic problems, schools must remain open, provided that the adults who follow children at home and at school absolutely comply with recommendations for prevention measures and that school facilities can be optimized in order to significantly reduce the spread of infection. In this regard, the universal use of face masks in addition to hand hygiene and safe distancing in schools, at least starting from the age of 6 years, seems extremely useful. Moreover, since the beginning of the COVID-19 outbreak the use of telemedicine to manage suspected SARS-CoV-2-infected individuals in the community has appeared to be an easy and effective measure to solve many paediatric problems and could represent a further support to schools .

**Conclusions:**

We think that schools must remain open, despite COVID-19 pandemic. However, several problems strictly related to school frequency and reduction of infectious risk must be solved before school attendance can be considered completely safe. A single more in-depth guideline agreed between countries with the same school problems could be very useful in eliminating doubts and fostering the compliance of students, teachers and non-teaching school staff reducing errors and misinterpretations.

## Background

At the beginning of the new coronavirus disease 2019 (COVID-19) pandemic, many governments around the world decided to close any type of school, including day cares and universities [1]. This was because it was thought that, as previously demonstrated by the influenza viruses, children were among the major spreaders of severe acute respiratory syndrome coronavirus 2 (SARS-CoV-2) and that by keeping them at home, a significant number of new COVID-19 cases could have been prevented. However, school closure during COVID-19 pandemic was strongly criticized by several experts [2] who reported that the assumptions on which this decision was based were not at all proven and that closing schools could have caused social, economic and health problems even more common and more severe than those due to COVID-19. The aims of this review are to discuss about children’s safety at school and the better strategies currently able to reduce the risk of SARS-CoV-2 infection at school.

## Can school closure influence COVID-19 pandemic?

A number of factors may cast doubts on the real usefulness of school to limit COVID-19 pandemic. Maintaining children at home in order to reduce the risk of them becoming infected and developing COVID-19 can be considered of poor clinical importance as children, when infected by SARS-CoV-2, generally remain asymptomatic or suffer from a mild disease that does not require hospital admission [3].

Moreover, school closure during the SARS and MERS epidemics was poorly effective in reducing morbidity and mortality, as was the experience of Taiwan, one of the very few countries where schools remained open during the first wave of the COVID-19 pandemic [4]. Studies that have monitored the epidemiology of SARS-CoV-2 before and after school re-opening when the first COVID-19 wave was considered over did not definitively solve the problem of the importance of school closure. In some countries, re-opening was not associated with any significant variations in COVID-19 incidence or related deaths [5, 6]. In contrast, in a study carried out in the USA, closure was associated with a significant reduction in SARS-CoV-2 circulation and related clinical problems [7]. However, it cannot be excluded that this positive effect was not strictly related to the school closure per se but could depend on several other concomitant factors, including the adherence of school workers and parents to the recommended preventive measures such as mask use, social distancing and frequent hand washing. Use of cloth face coverings in the U.S. population varied significantly during COVID-19 pandemic. Immediately after the first national recommendations were made, only approximately 60% of the enrolled adults reported the use of face masks [8]. On the contrary, 1 month later, in May, the prevalence of mask use increased to 76.4%. On the other hand, adherence to social distancing among people also seemed to be suboptimal. Finally, as most of the American people interviewed responded that they repeatedly wash their hands during the day to prevent COVID-19, a nonmarginal proportion of them (19%) do not regularly follow recommendations [9]. A further factor that may suggest that schools per se play a minor role in favouring COVID-19 diffusion is the evidence that, when general measures for infectious disease transmission were carefully followed, risk of transmission of SARS-CoV-2 from child to child during summer camps was several times lower than that of the general population.

Although reports of outbreaks of COVID-19 among children in schools and summer camps have been made [10], several studies have shown that children in educational setting and attending summer camps do not contribute significantly to COVID-19 diffusion [11]. On the other hand, previous experience has shown that school closure is systematically associated with the development of problems involving students, teachers and parents, particularly among populations with poor resources [2]. In children, opportunities for growth and development were dramatically reduced. Social isolation could lead to psychological limitations of much longer duration than the school closure itself. In some cases, nutrition could be compromised, as many children rely on free or discounted meals provided at schools. Furthermore, connections with students were frequently difficult, particularly when distance learning was not previously experienced and some parents had economic difficulties in activating e-learning.

The available experience highlights that in presence of an efficient contact tracing flexible protocols permit attendance of schools by children and adolescents without a significant increase in COVID-related morbidity and mortality.

## How schools may remain open with low risk for children

All the findings described above seem to confirm that, to ensure adequate learning and to avoid social and economic problems, schools must remain open, provided that the adults who follow children at home and at school absolutely comply with recommendations for prevention measures and that school facilities can be optimized in order to significantly reduce the spread of infection. Notably, the infrastructure of schools should be adapted, staff-to-pupil ratios should be addressed, and appropriate education on infectious disease prevention should be provided to ensure safe conditions. In this regard, the universal use of face masks in addition to hand hygiene and safe distancing in schools, at least starting from the age of 6 years, seems extremely useful [9]. Moreover, in order to maintain social distancing even outside the school avoiding crowding at the entrance and exit, changing or staggering school start and finish times for the various school classes must be established, public transportation network must be reorganized with increase in the number of available busses and underground trains. Where it is safe and appropriate to do, active travel must be strongly promoted, use of cars for longer journeys which cannot be accommodated on public transport must be accepted, and local businesses and employers must be extensively engaged to reduce other demand for public transport during peak school travel hours. Finally, students, particularly high-school students must be convinced to avoid gatherings and meetings outside school hours.

These data show that with appropriate education on infectious disease prevention and maintaining social distancing even outside the school, school may remain open with low risk of increase in SARS-CoV-2 circulation.

## Priorities for school strategy during COVID-19 pandemic

The above considerations highlight that particular attention must be paid to the use of preventive measure by students, teachers and non-teaching school staff. Guidelines on the use of masks in children, adolescents and teachers at school, as well as on how to manage classes in the presence of an infected student or teacher, are needed. Unfortunately, current recommendations, although accurately identify the subjects who can derogate from the use of masks, leave a lot of room for the discretionary power of teachers about the use of mask by other students.

The US Centers for Disease Prevention and Control indicate [12] that masks should not be placed on children younger than 2 years old, anyone who has trouble breathing or is unconscious, anyone who is incapacitated or otherwise unable to remove the mask without assistance. However, in some children in whom use of masks may be challenging such as in the case of younger students, those with severe asthma or other breathing difficulties and those with special educational or healthcare needs wearing masks is left to the judgment of the teachers. Similar problems arise for teachers themselves when they have chronic respiratory problems or must teach children who are deaf or hard of hearing. In the UK [13], it has been stated that the head teachers have the discretion to decide whether to ask staff or visitors to wear when social distancing cannot be safely managed. Similar recommendations are reported in Italy, where the Ministry of Health indicates that use of masks in children between 6 and 11 years of age has to be decided on the base of the local epidemiological situation, the characteristics of the child, his ability to wear the mask and the impact of this on the learning [14]. This can lead to different rules from one institution to another or even from one class to another and cause doubts about the most appropriate behaviour in all students and school staff with reduction in the use of masks even by those for whom use is clearly defined. This seems to indicate that as part of the comprehensive school strategy against SARS-CoV-2 (Table 1), the views of teachers, school-children and teenagers on the use of masks should be considered, including their perception of the risks. Special considerations should be given to situations where wearing a mask can significantly interfere with the learning process and negatively impact learning or other critical school activities (i.e., physical education, playing time). In addition, appropriate actions should be taken to ensure a sufficient supply of appropriate-sized masks for all school children and teenagers, and rules for waste management are needed to reduce the risk of disposing of masks in classrooms and playgrounds.

**Table 1:** Comprehensive and safe school strategy during COVID-19 pandemic

Strategy	Action
Universal use of face masks in children ≥6 years	Appropriate education of students and teachers
	Special consideration given to situations in which wearing a mask can significantly interfere with the learning process and negatively impact learning or other important critical school activities
	Sufficient supply of appropriate-sized masks
	Rules for waste management
Education on infectious disease prevention	Importance of hand hygiene
	Safe distance
Telemedicine	Activation of integrated software connected with hospitals
Organization	Adaptation of infrastructures
	Appropriate staff-to-pupil ratio

Furthermore, improvement of teachers and non-teaching staff knowledge in SARS-CoV-2 infection and COVID-19 characteristics is essential to minimize mistakes in mask use and identification of children with suspected infection. For educational issues and for the management of classes in the presence of infected cases, schools could derive relevant benefits from telemedicine. Since the beginning of the COVID-19 outbreak, the use of telemedicine to manage suspected SARS-CoV-2-infected individuals in the community has appeared to be an easy and effective measure to solve paediatric problems [15]. Use of telemedicine can be useful to comply with the recommendations of the Italian Pediatric Society that suggests that every measure be put into practice to separate the controls of healthy subjects from those of probably sick children [16]. The use of telemedicine in schools for health purposes is currently not supported by any real experience and experience between hospital and outpatient doctors cannot be considered *tout-court* applicable to the school. A prompt connection of each school with an expert in community health houses or in the hospital by telemedicine can allow easier identification of any schoolchild who has developed suspected symptoms of COVID-19 infection, allowing for a more rapid isolation and a prompt activation of diagnosis and treatment in cases at risk. The same approach with a connection from community health houses or the hospital and schools by telemedicine in support to health problems can be used in the future for other emergencies as well as for health promotion in schools.

## Conclusions

In conclusion, we think that schools must remain open, despite COVID-19 pandemic. However, several problems strictly related to school frequency and reduction of infectious risk must be solved before school attendance can be considered completely safe. A single more in-depth guideline agreed between countries with the same school problems could be very useful in eliminating doubts and fostering the compliance of students, teachers and non-teaching school staff reducing errors and misinterpretations.

## Data Availability

All included.

## Abbreviations

COVID-19: New coronavirus disease 2019
SARS-CoV-2: Severe acute respiratory syndrome coronavirus 2
